# Cryopreservation of Yak Semen: A Comprehensive Review

**DOI:** 10.3390/ani12243451

**Published:** 2022-12-07

**Authors:** Qudratullah Kalwar, Min Chu, Rashid Ali Korejo, Hidayatullah Soomro, Ping Yan

**Affiliations:** 1 Key Laboratory of Yak Breeding Engineering, Lanzhou Institute of Husbandry and Pharmaceutical Sciences, Chinese Academy of Agricultural Science, Lanzhou 730050, China; 2 Department of Animal Nutrition, Shaheed Benazir Bhutto University of Veterinary and Animal Sciences, Sakrand 67210, Pakistan

**Keywords:** yak, cryopreservation, semen

## Abstract

**Simple Summary:**

The yak is a multifunctional domesticated mammal that serves as a protein source for local herders and as a cultural and religious symbol. Recent worldwide climatic changes, namely, global warming, have directly impacted yak habitats; specifically, they have negatively influenced yak population sizes, as natural reproduction is not adequate to maintain a stable population. These conditions have caused researchers to investigate artificial yak reproduction. In this review, we cover numerous features of sperm cryopreservation in yaks and the many research efforts undertaken to enhance the cryopreservation process in yaks.

**Abstract:**

An urgent need to boost the sustainability and efficiency of animal production exists, owing to the growing global population. Enhancing the global fertility of animals, especially cattle, is essential to ameliorate this issue. Artificial insemination and sperm cryopreservation have a considerable and favorable influence on the quantity and quality of the cattle produced. Sperm cryopreservation is crucial for livestock production because it promotes and accelerates genetic diversity and the worldwide dispersion of animals with enhanced genetics. Owing to the importance of cryobiology in reproductive technologies, researchers are developing new approaches, and they are testing cryoprotectant drugs to enhance sperm cryosurvival. However, the viability of sperm after freezing is low and widely varies across breeding yaks. These faults are crucial because they impede advances in reproductive biotechnology and the study of mammalian gametes at a fundamental level. Using chemicals, researchers have developed and enhanced various extenders with varying degrees of efficiency to reduce cryodamage and oxidative stress. In this article, we review the cryopreservation of yak semen, the development of extenders, the difficulties faced during cryopreservation, and the evaluation of semen quality using various methodologies. This review might be helpful for researchers exploring semen cryopreservation in the future, as demand for enhanced cryopreservation exists to boost the post-thaw viability and fertility of sperm.

## 1. Introduction

Faced with an ever-expanding global population, increasing the efficiency and sustainability of animal food production is essential. Increasing the global fertility of yak, particularly cattle, is vital to resolve this issue. Improving the profitability of the cattle industry requires increased knowledge of reproductive biotechnology. Researchers have used artificial insemination (AI) and sperm cryopreservation to boost the efficiency and success rate of yak production [1], thereby accelerating genetic development and selection. Due to the fact that freezing and thawing damages many sperm cells, cryopreservation is not always successful [2].

Scientists initially introduced sperm cryopreservation in the 1600s [3]. In 1784, Italian scientist Lazzaro Spallanzani artificially inseminated three female dogs, resulting in the birth of three pups [4]. One hundred years later, in 1899, a Russian scientist named Ilya Ivanovich Ivanoff developed effective artificial insemination techniques for farm animals. In 1940, Phillips and Lardy discovered that egg yolks may protect sperm from cold shock during chilling; however, the use of this technique was neither widespread nor successful [5]. The results of another study showed that glycerol functions as a cryoprotectant at very low temperatures and throughout the freezing process [6]. Researchers have also used Na-citrate as a preservative to allow sperm to survive at low temperatures [7].

### 1.1. Yak Sperm Cryopreservation

Yaks breed on open ranges and in meadows in India’s trans-Himalayan and Himalayan regions. This highly sought-after cow breed flourishes in snow-bound, high-altitude locations where large-scale agriculture is unproductive, owing to a lack of fertile fields. Unlike other bovines, yaks supply milk, meat, fiber, wool, hide, fuel, and transportation in the Himalayas. Transhumance yak farming is associated with many of the same issues as other yak farming forms (e.g., poor management, reproductive problems, and dietary shortages), but it is less commonly practiced. Due to the fact that yaks are crucial to the subsistence of highlanders, development authorities are concerned about this lack of yak farming. Population loss and geographic isolation have increased the danger of inbreeding, which may reduce milk output and body size [8].

Yak semen is milky white, acidic (6.6–4.6.7), and 2–5 mL in volume [9]. Moreover, spermatozoa are present in concentrations ranging from 7.5 to 16.0 × 108/mL, 70 to 85 percent of which are gradually motile, 5.1 to 10 percent of which have an aberrant morphology, and 71.8 percent of which have intact acrosomes. At 15 degrees Celsius, the survival index and the general survival time of spermatozoa are 54 h and 18.72 h, respectively. Sperm has a resistance index of 5899, and semen has a viscosity of 1.17 centipoise, which is lower than that of semen from Bos taurus bulls (1.92 centipoise). Yak semen has a greater total nitrogen content (1334 mg/100 mL) than taurus semen (877 mg/100 mL) [10,11,12]. Its sperm morphology is similar to that of other bovines. A semen examination comprises an examination of sperm motility, viability, acrosomal integrity, and plasmatic membrane integrity and functioning [9].

Yak bulls can easily be trained for semen collection. However, due to a lack of knowledge about the genetic merit of young yak bulls, scientists collect the majority of semen from older, proven bulls that are regarded as superior. Sperm can be collected two times per week, and the season and age of the bull yak impact the volume and quality of the ejaculate [13]. The average volume obtained using an artificial vagina is 4.8 mL, with volumes collected from bulls aged two to eight years ranging from 2.6 to 6.0 mL [11]. AI and semen storage prevent inbreeding in yaks. Sperm cryopreservation helps to safeguard genetic resources and migrate species. Moving yaks for a natural service is time and cost-intensive and is particularly effective for mountainous yaks. In this review, we cover numerous features of sperm cryopreservation in yaks, which might be helpful for researchers exploring yak sperm cryopreservation in the future. Table 1 shows Literature survey regarding yak’s gametes cryopreservation.

### 1.2. Difficulties with Sperm Cryopreservation

Cryopreservation involves several phases, including lowering the temperature and decreasing the cells’ water content, dehydration, freezing, storage, and thawing. Sperm cells should be less vulnerable to harm during cryopreservation, owing to their low water content and strong membrane mobility. However, sperm integrity is reduced during cryopreservation due to changes in the membrane shape and function, as well as cell metabolism [14]. During the cooling and freezing phases, the cells are exposed to the following stressors, as outlined by Baust et al. [15]: (1) metabolic decoupling, an ionic discordant state, the activation of pro- and antiprotease enzymes and the resulting cellular acidosis, (2) the loss of energy, (3) membrane phase transition, (4) cytoskeleton destabilization, and (5) the production of free radicals or reactive oxygen species (ROS), all of which are detrimental to sperm during the free cooling process.

### 1.3. Molecular Challenges

Researchers often employ cell viability, motility, and morphology when analyzing the essential drivers of freezability, although modern methods have expanded the aspects of unique analyses. DNA integrity and chromatin structure are crucial components that influence the ability of sperm to survive cryopreservation, and they also aid in embryo development [16]. Freezing and thawing compromise the DNA integrity, exposing it to chemical and epigenetic modifications that might disrupt embryonic development and cause birth defects and other diseases [17]. This unfavorable process promotes chromatin instability, which results in DNA fragmentation in swine and avian sperm [18,19]. Cellular shrinkage and high ROS production levels [20], as well as mechanical stress on genomic regions due to increased chromatin compaction due to cell shrinkage [21], can all possibly cause DNA damage during cryopreservation [20,22]. Apoptosis and the excessive formation of reactive oxygen species (ROS) have been associated with sperm DNA cryoinjury and DNA damage, respectively [23]. This depends on whether the yak is fertile [24].

Sperm genome integrity and effective chromatin packing are critical for transmitting paternal DNA and epigenetic information to the egg. Mechanical, physiological, and chemical stressors may degrade chromatin. Recent studies show that freezing and thawing affect the chromatin structure of sperm. These alterations reduce sperm fertility but cause no other changes [25]. Polymerase and nuclease degrade DNA by attacking partially compacted chromatin [26]. Additionally, yaks’ low-quality sperm may induce infertility [27], and cryopreservation procedures may impact thawing [28,29]. When sperm are repeatedly frozen and thawed, DNA damage causes nuclear sperm changes [30,31].

### 1.4. Membrane Changes

Damage to the plasma membrane often occurs as a consequence of cryopreservation. Initially, researchers assumed that an imbalance in the membrane bilayer’s lipid content generated a cold shock [19]. During the chilling process, the membrane’s phospholipids become less mobile, resulting in a membrane that is more stiff and brittle. Phase transitions in lipid membranes occur in lipid phase separation, which leads to irreversible protein clustering [32].

### 1.5. Reactive Oxygen Species

Changes in mitochondrial membrane fluidity may result in the release of reactive oxygen species (ROS) and alterations in membrane potential during cryopreservation [33]. The superoxide anion (O), hydrogen peroxide (H_2_O_2_), and nitric oxide (NO) all contribute to intracellular communication, sperm capacitation, and acrosome activity [34]. Capacitation and responses to acrosomal stimuli are essential for sperm activity, but in high quantities, these chemicals are toxic to sperm and impair their capacity to reproduce. The exact synthesis and function of reactive oxygen species (ROS) in sperm are not yet completely known [1]. These compounds have been associated with protein inactivation, lipid peroxidation, and DNA damage as a result of a lack of molecular oxygen reduction.

### 1.6. Molecular Markers of Sperm Freezability

Seminal plasma and sperm proteins promote sperm survival, fertilization, and energy metabolism [30]. Researchers found a link between yaks’ capacity to freeze and protein levels in sperm and seminal plasma. These proteins inhibit sperm migration by attaching to plasma membrane phospholipids. Poor cryoprotectant yak sperm had decreased HSP90 levels [31,35]. Low HSPA8 levels in a freezing medium reduce sperm survival after thawing, whereas larger levels increase plasma membrane integrity.

Mostek and colleagues [36] found that sperm protein carbonylated after cryopreservation, and this process involved NADH dehydrogenase, ropporin-1, actin-related protein T2, outer dense fiber protein 2, glutathione S-transferases, triosephosphate isomerase, and cilia. L-PGDS was linked to yak sperm’s poor freezing stability. High-freezing semen had higher acidic seminal fluid protein (aSFP) levels, and aSFP supporters say it reduces lipid peroxidation to preserve sperm from oxygen stress [37]. 

**Table 1:** Literature survey regarding yak’s gametes cryopreservation.

Tittle	Summary	References
Effects of cryopreservation on enzyme activities of wild yak sperm.	When wild yak sperm was cryopreserved, enzyme activity decreased. The descent degree increased as the cryopreservation time was extended.	[38]
The effect of freezing on yak sperm cryosurvival.	After a 4-h equilibration interval, yak semen exhibited a greater cryosurvival when frozen in tris extender with 6.4 percent glycerol and 20% egg yolk.	[39]
Current sperm cryopreservation status: why is it not better?	Features of sperm cryopreservation are mirrored by capacitation events and assess the potential roles of sperm membrane cholesterol, reactive oxygen species, and seminal plasma as mediators of cryopreservation effects on sperm function.	[40]
Heparin-induced and caffeine- or ouabain-supplemented capacitation of frozen-thawed yak (Bos grunniens) spermatozoa.	Caffeine synergistically increases yak sperm capacitation with heparin, whereas ouabain does not synergistically boost yak sperm capacitation with heparin.	[41]
Developmental competence of frozen-thawed yak (Bos grunniens) oocytes followed by in vitro maturation and fertilization.	To cryopreserve yak oocytes in French straws, a mixture of EG and DMSO or EG, Ficoll, and sucrose can be utilized.	[42]
Effects of orvus paste on the motility and viability of yak (Bos grunniens) epididymal and ejaculated spermatozoa after freezing and thawing.	To preserve yak spermatozoa, 0.75 percent OEP is effective.	[43]

## 2. Extender Development

To reduce cryodamage and increase post-thaw vitality, researchers have produced several extenders. To cryopreserve cattle, buffalo, and pig sperm, scientists often use extenders based on 20% egg yolk [44]. Moreover, the high-density lipoproteins and minerals found in egg yolk granules impede sperm cell respiration and decrease their motility; additionally, egg yolk also protects cells from harm during cryopreservation [45]. However, the sperm membrane is shielded from harm by the low-density lipoproteins (LDL) of the egg yolk during freezing and thawing [46]. Moreover, plant-based extenders prevent disease transmission in frozen sperm [47,48]. The results of one study on liposome-based extenders showed that they were more effective than animal- and plant-based extenders [49], whereas the authors of another study found no significant variations in the post-thaw properties of soy-lecithin- and skim-milk-extended goat sperm [50,51]. Muiño et al. [52] used Biladyl R, which includes tris egg yolk, to preserve eggs. Moreover, the results of another study revealed that tris-egg-yolk-based extenders are more effective than plant-based extenders at preserving sperm [53].

### 2.1. Cryoprotectant Supplementation of Extenders

High levels of penetrating cryoprotectants (CPAs) can eliminate ice formation during the cryopreservation of cells, tissues, and organs to cryogenic temperatures. In addition, nonpenetrating and penetrating cryoprotectants protect sperm cells against ice crystallization’s physical and chemical obstacles. Moreover, nonpenetrating cryoprotectants, such as polymers, are preferable in vitrification, whereas penetrating cryoprotectants, such as sugars, lower the end product toxicity [54].

Glycerol is the most common osmotic stimulant in bovine sperm [55]. Skim-milk-based extenders have 8% CPA, whereas tris-egg-yolk-based extenders have 6–7% CPAs [56]. Sperm cells lose lipids and phospholipids during cryopreservation [57]. The extender’s fatty acid content controls fluidity, which impacts membrane freezing. Moreover, Docosahexaenoic acid enhances sperm vitality following freezing [57].

Egg-yolk-based extenders with 8% coconut oil, 5 ng/mL arachidonic acid, and 20 ng/mL linoleic acid increase sperm quality after cryopreservation [58,59,60,61]. Iodixanol may help make density gradient centrifugation more efficient. OptiPrepTM iodixanol enhances yak sperm motility following freezing. Moreover, iodixanol may maintain sperm membranes by avoiding ice crystal formation [62,63].

### 2.2. Antioxidant Supplementation of Extenders

Antioxidants inhibit reactive oxygen species (ROS) and lipid peroxidation; in addition, they protect sperm cells from oxidative damage [64]. Conversely, glutathione protects yak sperm against free radical damage and boosts motility, plasma membrane integrity, and cell survival. Resveratrol protects sperm against superoxide, hydroxyl, and metal-induced radicals [64]. Vitamin E increases prostate motility and membrane integrity [65]. Moreover, bovine sperm cryopreservation is ineffective without endogenous antioxidants. Bovine serum albumin (BSA) protects the cell shape and acrosome integrity and boosts catalase activity [66]. Additionally, carnitine and inositol increase sperm motility, DNA protection, and acrosome integrity [67].

Plant-derived antioxidants are less cytotoxic than synthetics, meaning that adding 0.75 percent green tea extract to cryopreserved spermatozoa may increase sperm motility [68]. In addition, selenium in a semen extender increased cryopreserved sperm integrity [69]. Moreover, spirulina maxima extract (SME), a microalga, increases sperm motility, morphology, and ROS production [70]. Trehalose, an antioxidant sugar, may protect sperm cells from oxidative and cold shock damage, and it enhances sperm motility and membrane integrity [71].

### 2.3. Vitamins and Other Supplementations of Extenders

Antioxidant-rich vitamins and substances prevent cryodamage and increase sperm quality after thawing. Vitamin C may protect the organism against oxidative stress and metabolic activity by neutralizing free radicals. Ascorbate free radicals’ electron-transporting capacity reduces oxidative stress. In addition, one study reported that supplementing ejaculate with vitamin C increased sperm quality and decreased defective sperm [72]. Likewise, post-thaw motility and integrity increased with vitamin C supplementation to sperm extenders [73]. Administering ascorbic acid to yaks increased their ejaculate volume and sperm concentration, changing the scrotal circumference, response time, and total sperm production [74,75,76].

Herbal extracts and supplements may potentially affect animal reproduction. El-Sheshtawy and El-Nattat found that silymarin increased the viability of frozen yak sperm. Daghigh-Kia et al. [76] added rosemary extract, glutathione (GSH), or both help to preserve yak sperm, and found that the rosemary extract increased the yak sperm’s post-thaw properties. Table 2 and Table 3 show literature survey regarding Extender development and composition of extender for sperm cryopreservation. 

## 3. Techniques to Evaluate Sperm Quality

To determine the relationship between cell shape at the molecular and cellular levels and cell function, one must undertake comprehensive research on sperm using many integrated methods. To capture the cell, genetic, functional, and epigenetic material of sperm, for instance, researchers should utilize the most applicable, advanced, and standardized methods. To find precise determinants of sperm motility, viability, membrane function, and mitochondrial activity, one must use enhanced cryopreservation methods.

### 3.1. Microscopy

Researchers can analyze motility, shape, membrane integrity, and concentration using light microscopy. Fluorochromes have caused fluorescent microscopy to become popular in biological and reproductive studies. Moreover, researchers use fluorescence microscopy to examine sperm, membrane, acrosome, and chromatin viability. Researchers can use fluorescence microscopy to test sperm DNA, membranes, and lectins. Furthermore, researchers can use fluorescence microscopy to detect sperm viability using SYBR-14 (DNA binding) and PI (membrane permeable) stains. Additionally, they can use TUNEL and fluorescence microscopy to identify apoptosis.

Scientists can use confocal microscopy to examine all three sperm parts. They may use this approach to analyze sperm cell surface proteins [91,92] and cytoskeletal proteins [92,93]. Additionally, they can use mitochondrial microscopy to monitor sperm; however, it lacks the quantification of many parameters. Thus, researchers may use it to analyze mitochondrial activity at the single cell level and to track sperm with active mitochondria [94,95]. Additionally, this approach allows researchers to measure sperm lipid peroxidation and ROS [95].

Researchers primarily use transmission electron microscopes and scanning electron microscopes in labs. A scanning electron microscope (SEM) can magnify sperm cell surfaces, but it lacks resolution. Additionally, researchers can use SEM to determine if cryopreservation changes sperm morphology. Transmission electron microscopes (TEM) help researchers investigate sperm morphology and its function [96]. Additionally, researchers can use an electron microscope (EM) to identify aberrant sperm [97].

Scientists may examine the morphology, structural integrity, and structure of yak sperm using holographic microscopy [98,99,100]. This high-throughput technology employs image reconstruction to track sperm heads and tails in 3D [101]. The use of a laser to induce direct inelastic light scattering enables scientists to use Raman spectroscopy on the scattered photons. This frequency offers reliable information about normal and abnormal human sperm components, such as proteins and DNA [102,103]. Researchers can use this approach to analyze bovine and human sperm cells [104]. Furthermore, they can use Raman spectroscopy and holographic microscopy to evaluate sperm morphology and biochemistry [105,106].

### 3.2. Computer-Assisted Sperm Analysis (CASA)

The CASA system, introduced in 1980, is a software-based computer technique used to measure sperm motility and kinematics. Using a limited field, pictures of moving sperm are transformed into video images with varied acquisition speeds (frames s-1, Hz). Scannable images utilizing a dark field or negative high-phase contrast track the movements of individual sperm while accounting for pixel intensity and the head [107,108]. CASA provides sperm motility statistics (progressive and total) and kinematic parameters (velocity, linearity, and lateral displacement), which characterize the sperm’s trajectory during the investigation. The system uses straight line (VSL), curved (VCL), average path (VAP), and linearity of forward progress (LIN) (ALH) to measure the sperm movement velocity. Existing CASA systems more effectively analyze sperm viability, concentration, morphology, and DNA fragmentation because of their high-quality hardware and open-source software [109,110].

### 3.3. Flow Cytometry

Flow cytometry is the quickest and most precise approach one can use to study many cells. Researchers using flow cytometry study single cells with spermatozoon-like properties. They can use fluidic, optical, and electromechanical sensors to detect fluid fluorescence [111].

For this procedure, researchers fill the instrument’s flow cell with sperm cells or fluorescently tagged particle samples. Then, the instrument absorbs the sperm cells, and it uses two lenses to measure the fluorescent band widths. The fluorescence is subtracted from the overall fluorescence intensity to account for the spermatozoon characteristics. Researchers can use fluorescence-activated flow cytometry (FACS) [112,113] to separate and purify cells. The use of these repeatable, accurate, sensitive, and precise techniques may cause the instrument to detect the fluorescence emission without sorting. This approach allows researchers to gather data on subpopulations and different populations, such as sperm. Researchers can use flow cytometry to analyze many sperm organelles.

Viable and nonviable sperm are differentiated based on the membrane’s molecular structure and function. Temperature and osmotic stress reduce sperm viability during cryopreservation. EH, PI, Yo-Pro-1, and Hoechst 33,258 may excite lasers alone or in combination [113,114]. Propidium iodide (PI) passes through the plasmalemma of nonviable sperm when activated by a 488 nm laser [115]. SYBR14 and PI illuminate the active cell nuclei. Using various dye combinations, researchers can use this staining approach to determine acrosome integrity or mitochondrial activity. Researchers have used SYBR14-PI staining to study cryopreservation’s effect on sperm viability in bees, horses, cattle, and fish. Using Yo-Pro-1, one can measure membrane permeability at 509 nm [116]. Additionally, one can utilize a Yo-Pro-1 and Ethidium homodimer to examine cryopreserved sperm membrane integrity [117]. SYBR-14 and PI detect membrane breakdown earlier than Yo-Pro-1 and PI [118]. When bound to nucleic acid, Hoechst 33,258 fluoresces blue at 462 nm. Thus, scientists can use Hoechst 33258’s green–red signal with other probes to detect viable sperm [119].

Flow cytometry requires fluorescent isothiocyanate-labeled lectin probes to identify acrosomes (FITC). Attached to a damaged spermatozoon, the PNA label exhibits a green fluorescence [120]. When coupled with FITC-PSA and PI, researchers can use it to assess plasma membrane and acrosomal integrity. PNA-FITC staining identifies acrosomes more accurately than PSA-FITC. Researchers can use FITC-PNA and PI staining to identify sperm quality and acrosome integrity [121,122].

### 3.4. Oxidative Stress Analysis

Oxidative stress reduces sperm’s fertility. OH radicals, superoxide anion ions, and nonradical hydrogen peroxide ions are ROS [123]. A cold shock increases ROS and lipid peroxidation during cryopreservation and thawing. Researchers can use flow cytometry to identify ROS and oxidative species more accurately. H2DCFDA measures intercellular hydrogen peroxide (H2O2) levels. Nonfluorescent intercellular H2DCFDA may pass the cell membrane and stay. The 2′,7′-dichlorofluorescein (DCF) emits at 517–527 nm [124,125]. Superoxide oxidizes the reduced form, producing a 610 nm red fluorescence. Researchers can use this probe to analyze ROS in sperm when using it with cell viability indicators. CM-H2DCFDA measures hydrogen peroxide more accurately than CM-H2DCFDA (HCFDA). Esterases and a thiol-reactive chloromethyl group break down acetates to create H2DCF. H2O^2^ oxidizes H2DCF to DCF at 525 nm [126]. This test determines the yak sperm oxidative stress. MitoSOX Red is a probe that can detect superoxide in mitochondria and cells [127].

The chromatin form predicts sperm quality and fertility. AO causes a metachromatic shift from green to red fluorescence in SCSA [128]. When the AO attaches to double-stranded DNA, green fluorescence ensues. By analyzing spermatozoon fluorescence, researchers can evaluate DNA fragmentation and chromatin structure. In addition to TUNEL, researchers can use flow cytometry to assess sperm DNA damage. Terminal deoxynucleotidyl transferase incorporates deoxyuridine triphosphate nucleotides into DNA breaks at their 3′ hydroxyl ends. Researchers can employ SCSA and TUNEL [129,130] with flow cytometry to assess sperm fragmentation.

Sex sorting requires high-speed flow cytometers and the correct methods. Researchers can use flow cytometry to differentiate sperm’s X and Y chromosomes [129]. Previously, researchers have used a fluorescence detector to detect each spermatozoon’s fluorescence; according to the chromosomal fluorescence, they deemed the droplets as positively or negatively charged, and then they split the DNA into X and Y tubes with a charged plate [130].

We will need bioinformatics or mathematical biology to understand more about sperm quality and viability after freezing. Functional genomics (transcriptomics, proteomics) and DNA methylation and dynamics may benefit AI techniques. Scientists may create novel biomarkers such as microRNAs, lipids, and other tiny molecules, or epigenomic indicators such as proteins and lipids, to better understand spermatogenesis and sperm quality.

## 4. Conclusions

The use of artificial insemination (AI) and semen cryopreservation may be one of the most effective strategies to address the inbreeding problem in yaks. Artificial insemination and sperm cryopreservation have a remarkably positive impact on cattle production and product quality. Sperm cryopreservation enables animals to relocate to distant areas and conserve their genetic resources. Sperm from the best-breeding bulls can be used to inseminate thousands of animals around the world using cryopreserved sperm and artificial insemination. Although bull sperm cryopreservation has progressed beyond that of other species, considerable gaps in knowledge and technology still exist. This article provides details regarding yak semen cryopreservation, extenders development, the difficulties faced during cryopreservation, and how to evaluate semen quality using various methodologies. This review might be helpful for future studies that explore semen cryopreservation and the enhancement of the production potential of yak.

## Figures and Tables

**Table 2:** Extender development for sperm cryopreservation (Ugur et al. [77]).

Supplement	Functions or Effects	References
**CRYOPROTECTANTS**		
Egg yolk	When frozen, low-density lipoproteins in egg yolk bind to cell membrane and create an interfacial coating.	[78,79]
Milk	Sperm cells are shielded from cryodamage by the protein part of skim milk.	[1]
Glycerol	Responsible for lipid and protein rearrangement in the membrane.	[54]
Ethylene glycol	Increase dehydration at lower temperatures to reduce intracellular ice formation.	[80]
Dimethyl sulfoxide		
Propylene Glycol		
Trehalose	By substituting for water, replace the bound water surrounding macromolecules and protectively hydrate those macromolecules.	[81]
Polyols	Create hydrogen bonds with lipid membrane; therefore, sperm membrane is stabilized at low temperatures.	[2]
Butylated hydroxytoluene	Increases fluidity of the membrane and decreases activity of the lipid peroxyl radicals, which increase motility, acrosomal integrity, and membrane integrity.	[82]
**ANTIOXIDANTS**		
Glutathione	Supplementing with glutathione increases vitality, plasma membrane stability, and motility.	[63]
Resveratrol	Removes radicals produced by metals, hydroxyl, and superoxide. In light of this, it guards against ROS damage to sperm chromatin and membranes.	[83]
Bovine Serum Albumin	Increases the catalase activity and aids in maintaining the integrity of the acrosome and the shape of the cell.	[64]
Methionine	Keeps sperm morphology normal.	[65]
Carnitine	Enhances sperm motility, acrosome integrity, and DNA damage prevention.	[66]
**VITAMINS**		
Vitamin C	Post-thaw motility and the percentage of intact plasma are both increased by vitamin C intake.	[72]
Vitamin E	Positively influences membrane integrity, membrane potential, and sperm motility.	[9]

**Table 3:** Composition of extender for yak frozen semen [84].

Basic Solution (mL)	Egg Yolk (mL)	Glycerol (mL)	Post-Thawing Motility	Conception Rate (%)	References
12% sucrose 75	20	5	0.55 ± 0.14 0.36 ± 0.04	13/20 (65) 39/52 (5)	[85,86]
12% sucrose 75	20	5	0.43	49/61 (80.3)	[87]
12% sucrose 75	20	5	0.4 ± 0.11		[88]
12% sucrose 75	20	5	0.4 ± 0.5		[89]
Skimmed milk 80	20	3	0.4 ± 0.5	0.55+/−0.07	[85]
3.97% sodium citrate dihydrate	20	7	0.4 ± 0.5	84/114 (73.7)	[89]
12% lactose 75	20	5	0.4 ± 0.5		
7.5% glucose 75	20	5	0.38 ± 0.09		[90]
11% lactose 75	20	5	0.4 ± 0.1		

## References

[1] Medeiros C., Forell F., Oliveira A., Rodrigues J. (2002). Current status of sperm cryopreservation: Why isn't it better?. Theriogenology.

[2] Bailey J.L., Bilodeau J.F., Cormier N. (2000). Semen cryopreservation in domestic animals: A damaging and capacitating phenomenon. J. Androl..

[3] Sherman J. (1964). Low temperature research on spermatozoa and eggs. Cryobiology.

[4] Ombelet W., Van Robays J. (2015). Artificial insemination history: Hurdles and milestones. Facts, Views Vis. ObGyn.

[5] Phillips P.H., Lardy H.A. (1940). A yolk-buffer pabulum for the preservation of Yak semen. J Dairy Sci..

[6] Polge C., Smith A.U., Parkes A.S. (1949). Revival of Spermatozoa after Vitrification and Dehydration at Low Temperatures. Nature.

[7] Salisbury G., Fuller H., Willett E. (1941). Preservation of Bovine Spermatozoa in Yolk-Citrate Diluent and Field Results from its Use. J. Dairy Sci..

[8] Lu Z.L. (2000). Reproduction and Conservation of Wild Yak. Recent Advances in Yak Reproduction.

[9] Zhang Z.W. (2000). Semen Characteristics and Artificial Insemination in Yak. Recent Advances in Yak Reproduction.

[10] Zhang Y. (1989). Experimental reports on the manufacture of frozen semen of yak. J. China Yak.

[11] Wang M.Q., Lu Z.L. (1990). Studies on reproductive characteristics of male yak (domestic and wild). J. China Yak.

[12] Du F.S., Arlongga, Ma S.R. (1994). Experimental report on conception in strange land and frozen semen making of Jiulong yak. J. China Yak.

[13] Zhan Y. The relation ship between season and age of stud yak bull in Dangxun. Proceedings of the 1st International Congress on Yak.

[14] Hammerstedt R.H., Graham J.K., Nolan J.P. (1990). Cryopreservation of mammalian sperm: What we ask them to survive. J. Androl..

[15] Baust J.G., Gao D. (2009). Cryopreservation. Organogenesis.

[16] Agarwal A., Varghese A.C., Sharma R.K., Park-Sarge O.K., Curry T. (2009). Markers of Oxidative Stress and Sperm Chromatin Integrity. Molecular Endocrinology. Methods in Molecular Biology (Methods and Pro-Tocols).

[17] Lewis S.E.M., Aitken R.J. (2005). DNA damage to spermatozoa has impacts on fertilization and pregnancy. Cell Tissue Res..

[18] Fraser L., Strzeżek J. (2007). Is there a relationship between the chromatin status and DNA fragmentation of boar spermatozoa following freezing–thawing?. Theriogenology.

[19] Gliozzi T.M., Zaniboni L., Cerolini S. (2011). DNA fragmentation in chicken spermatozoa during cryopreservation. Theriogenology.

[20] Watson P.F. (1995). Cooling of Spermatozoa and Fertilizing Capacity. Reprod. Domest. Anim..

[21] McCarthy M.J., Baumber J., Kass P.H., Meyers S.A. (2010). Osmotic Stress Induces Oxidative Cell Damage to Rhesus Macaque Spermatozoa1. Biol. Reprod..

[22] Bogle O.A., Kumar K., Attardo-Parrinello C., Lewis S.E.M., Estanyol J.M., Ballescà J.L., Oliva R. (2016). Identification of protein changes in human spermatozoa throughout the cryopreservation process. Andrology.

[23] Kopeika J., Thornhill A., Khalaf Y. (2015). The effect of cryopreservation on the genome of gametes and embryos: Principles of cryobiology and critical appraisal of the evidence. Hum. Reprod. Update.

[24] Johnston S.D., Satake N., Zee Y., López-Fernández C., Holt W.V., Gosálvez J. (2012). Osmotic stress and cryoinjury of koala sperm: An integrative study of the plasma membrane, chromatin stability and mitochondrial function. Reproduction.

[25] Kumar D., Kumar P., Singh P., Yadav S.P., Sarkar S.K., Bharadwaj A., Yadav P.S. (2014). Characteristics of frozen thawed semen in predicting the fertility of buffalo Yaks. Indian J. Anim. Sci..

[26] Silva P., Gadella B. (2006). Detection of damage in mammalian sperm cells. Theriogenology.

[27] Khalil W.A., El-Harairy M.A., Zeidan A.E., Hassan M.A., Mohey-Elsaeed O. (2018). Evaluation of bull spermatozoa during and after cryopreservation: Structural and ultrastructural insights. Int. J. Veter.-Sci. Med..

[28] Anzar M., He L., Buhr M.M., Kroetsch T.G., Pauls K.P. (2002). Sperm Apoptosis in Fresh and Cryopreserved Bull Semen Detected by Flow Cytometry and Its Relationship with Fertility1. Biol. Reprod..

[29] Castro L.S., Hamilton T.R.D.S., Mendes C.M., Nichi M., Barnabe V.H., Visintin J.A., Assumpção M.E.O.A. (2016). Sperm cryodamage occurs after rapid freezing phase: Flow cytometry approach and antioxidant enzymes activity at different stages of cryopreservation. J. Anim. Sci. Biotechnol..

[30] Hammadeh M.E., Dehn C., Hippach M., Zeginiadou T., Stieber M., Georg T., Rosenbaum P., Schmidt W. (2001). Comparison between computerized slow-stage and static liquid nitrogen vapour freezing methods with respect to the deleterious effect on chromatin and morphology of spermatozoa from fertile and subfertile men. Int. J. Androl..

[31] Moura A.A., Memili E. (2016). Functional aspects of seminal plasma and sperm proteins and their potential as molecularmarkers of fertility. Anim. Reprod..

[32] Zhang X.-G., Hu S., Han C., Zhu Q.-C., Yan G.-J., Hu J.-H. (2015). Association of heat shock protein 90 with motility of post-thawed sperm in bulls. Cryobiology.

[33] De Leeuw F.E., Chen H.-C., Colenbrander B., Verkleij A.J. (1990). Cold-induced ultrastructural changes in bull and boar sperm plasma membranes. Cryobiology.

[34] Said T.M., Gaglani A., Agarwal A. (2010). Implication of apoptosis in sperm cryoinjury. Reprod. Biomed. Online.

[35] Aitken R.J. (1995). Free radicals, lipid peroxidation and sperm function. Reprod. Fertil. Dev..

[36] Holt W., Del Valle I., Fazeli A. (2015). Heat shock protein A8 stabilizes the bull sperm plasma membrane during cryopreservation: Effects of breed, protein concentration, and mode of use. Theriogenology.

[37] Mostek A., Dietrich M.A., Słowińska M., Ciereszko A. (2017). Cryopreservation of bull semen is associated with carbonylation of sperm proteins. Theriogenology.

[38] Einspanier R., Krause I., Calvete J., Töfper-Petersen E., Klostermeyer H., Karg H. (1994). Bovine seminal plasma ASFP: Localization of disulfide bridges and detection of three different isoelectric forms. FEBS Lett..

[39] Jinxing L., Ping Y., Xian G., Yufeng Z., Yuni Y. (2010). Effects of Cryopreservation on Enzyme Activities of Wild Yak Sperm. https://europepmc.org/article/cba/646855.

[40] Deori S. (2018). The effect of freezing on cryosurvival of yak sperm. SAARC J. Agric..

[41] Zhang R., Chu M., Chen Y., Yan P. (2022). Heparin-induced and caffeine or ouabain supplemented capacitation of frozen-thawed yak (Bos grunniens) spermatozoa. Reprod. Domest. Anim..

[42] Niu H.-R., Zi X.-D., Xiao X., Xiong X.-R., Zhong J.-C., Li J., Wang L., Wang Y. (2014). Developmental competence of frozen-thawed yak (Bos grunniens) oocytes followed by in vitro maturation and fertilization. Cryobiology.

[43] Shimazaki M., Sambuu R., Sato Y., Do L.T.K., Tanihara F., Taniguchi M., Otoi T. (2015). Effects of Orvus Es Paste on The Motility And Viability Of Yak (Bos Grunniens) Epididymal And Ejaculated Spermatozoa After Freezing And Thawing. Cryoletters.

[44] Bathgate R., Maxwell W., Evans G. (2006). Studies on the Effect of Supplementing Boar Semen Cryopreservation Media with Different Avian Egg Yolk Types on in Vitro Post-thaw Sperm Quality. Reprod. Domest. Anim..

[45] Moussa M., Martinet V., Trimeche A., Tainturier D., Anton M. (2002). Low density lipoproteins extracted from hen egg yolk by an easy method: Cryoprotective effect on frozen–thawed bull semen. Theriogenology.

[46] Amirat L., Tainturier D., Jeanneau L., Thorin C., Gérard O., Courtens J.L., Anton M. (2003). Bull semen in vitro fertility after cryopreservation using egg yolk LDL: A comparison with Optidyl®, a commercial egg yolk extender. Theriogenology.

[47] Murphy E., O’Meara C., Eivers B., Lonergan P., Fair S. (2018). Comparison of plant- and egg yolk-based semen diluents on in vitro sperm kinematics and in vivo fertility of frozen-thawed bull semen. Anim. Reprod. Sci..

[48] Yodmingkwan P., Guntaprom S., Jaksamrit J., Lertchunhakiat K. (2016). Effects of Extenders on Fresh and Freezing Semen of Boer Goat. Agric. Agric. Sci. Procedia.

[49] Vidal A.H., Batista A.M., da Silva E.C.B., Gomes W.A., Pelinca M.A., Silva S.V., Guerra M.M.P. (2013). Soybean lecithin-based extender as an alternative for goat sperm cryopreservation. Small Rumin. Res..

[50] Naz S., Umair M., Iqbal S. (2018). Comparison of Tris egg yolk-based, Triladyl^®^and Optixell^®^extender on post-thaw quality, Kinematics and in vivo fertility of Nili Ravi Buffalo *(Bubalus bubalis)* bull spermatozoa. Andrologia.

[51] Muiño R., Fernández M., Peña A. (2007). Post-thaw Survival and Longevity of Bull Spermatozoa Frozen with an Egg Yolk-based or Two Egg Yolk-free Extenders after an Equilibration Period of 18 h. Reprod. Domest. Anim..

[52] Crespilho A., Filho M.S., Dell'Aqua J., Nichi M., Monteiro G., Avanzi B., Martins A., Papa F. (2012). Comparison of in vitro and in vivo fertilizing potential of bovine semen frozen in egg yolk or new lecithin based extenders. Livest. Sci..

[53] Oldenhof H., Friedel K., Sieme H., Glasmacher B., Wolkers W.F. (2010). Membrane permeability parameters for freezing of stallion sperm as determined by Fourier transform infrared spectroscopy. Cryobiology.

[54] Barbas J.P., Mascarenhas R.D. (2008). Cryopreservation of domestic animal sperm cells. Cell Tissue Bank..

[55] Best B.P. (2015). Cryoprotectant Toxicity: Facts, Issues, and Questions. Rejuvenation Res..

[56] Purdy P. (2006). A review on goat sperm cryopreservation. Small Rumin. Res..

[57] Vishwanath R., Shannon P. (2000). Storage of bovine semen in liquid and frozen state. Anim. Reprod. Sci..

[58] Sarmah B., Kaker M., Razdan M. (1984). Effect of cold shock and freezing on loss of total lipids and phospholipids of buffalo spermatozoa (Bubalusbubalis). Theriogenology.

[59] Takahashi T., Itoh R., Nishinomiya H., Katoh M., Manabe N. (2011). Effect of Linoleic Acid Albumin in a Dilution Solution and Long-term Equilibration for Freezing of Bovine Spermatozoa with Poor Freezability. Reprod. Domest. Anim..

[60] Tarig A., Wahid H., Rosnina Y., Yimer N., Goh Y., Baiee F., Khumran A., Salman H., Ebrahimi M. (2017). Effect of different concentrations of egg yolk and virgin coconut oil in Tris-based extenders on chilled and frozen-thawed bull semen. Anim. Reprod. Sci..

[61] Saragusty J., Gacitua H., Rozenboim I., Arav A. (2009). Protective effects of iodixanol during bovine sperm cryopreservation. Theriogenology.

[62] Kaka A., Wahid H., Rosnina Y., Yimer N., Khumran A., Sarsaifi K., Behan A.A., Kaka U., Ebrahimi M. (2015). α-Linolenic acid supplementation in BioXcell® extender can improve the quality of post-cooling and frozen-thawed bovine sperm. Anim. Reprod. Sci..

[63] Sanocka D., Kurpisz M. (2004). Reactive oxygen species and sperm cells. Reprod. Biol. Endocrinol..

[64] Peña F., Johannisson A., Wallgren M., Martinez H.R. (2003). Antioxidant supplementation in vitro improves boar sperm motility and mitochondrial membrane potential after cryopreservation of different fractions of the ejaculate. Anim. Reprod. Sci..

[65] Sariözkan S., Tuncer P.B., Bucak M.N., Ulutaş P.A. (2009). Influence of Various Antioxidants on Microscopic-Oxidative Stress Indicators and Fertilizing Ability of Frozen-Thawed Bull Semen. Acta Veter. Brno.

[66] Bucak M.N., Tuncer P.B., Sarıözkan S., Başpınar N., Taşpınar M., Çoyan K., Bilgili A., Akalın P.P., Büyükleblebici S., Aydos S. (2010). Effects of antioxidants on post-thawed bovine sperm and oxidative stress parameters: Antioxidants protect DNA integrity against cryodamage. Cryobiology.

[67] Khan H., Khan M., Qureshi M.S., Ahmad S., Gohar A., Ullah H., Ullah F., Hussain A., Khatri P., Shah S.S.A. (2017). Effect of Green Tea Extract (Camellia sinensis) on Fertility Indicators of Post-Thawed Bull Spermatozoa. Pak. J. Zool..

[68] Mizera A., Kuczaj M., Szul A. (2019). Impact of the *Spirulina maxima* extract addition to semen extender on bovine sperm quality. Ital. J. Anim. Sci..

[69] Hu J.-H., Zan L.-S., Zhao X.-L., Li Q.-W., Jiang Z.-L., Li Y.-K., Li X. (2010). Effects of trehalose supplementation on semen quality and oxidative stress variables in frozen-thawed bovine semen1. J. Anim. Sci..

[70] Zubair M., Ali M., Ahmad M., Sajid S.M., Ahmad I., Gul S.T. (2015). Effect of Selenium and Vitamin E on Cryopreservation of Semen and Reproductive Performance of Animals (A Review). J. Entomol. Zool. Stud..

[71] Mittal P.K., Anand M., Madan A.K., Yadav S., Kumar J. (2014). Antioxidative capacity of vitamin E, vitamin C and their combination in cryopreserved Bhadavari bull semen. Veter-World.

[72] Andrabi S.M.H., Ansari M., Ullah N., Afzal M.N. (2008). Effect of NonEnzymatic Antioxidants in Extender on Post-Thaw Quality of Buffalo (Bubalus Bubalis) Spermatozoa. Pak. Vet. J..

[73] Gabr A.A.-W., El Basuini M. (2018). Effect of tonophosphan, zinc oxide, and ascorbic acid on semen, sexual desire, and the fertility rate of Egyptian buffalo bulls. Ann. Agric. Sci..

[74] Das S.K., Vasudevan D. (2006). Protective effects of silymarin, a milk thistle (Silybium marianum) derivative on ethanol-induced oxidative stress in liver. Indian, J. Biochem. Biophys..

[75] El-Sheshtawy R., El-Nattat W. (2017). Impact of silymarin enriched semen extender on bull sperm preservability. Asian Pac. J. Reprod..

[76] Daghigh-Kia H., Olfati-Karaji R., Hoseinkhani A., Ashrafi I. (2014). Effect of rosemary (Rosmarinus officinalis) extracts and glutathione antioxidants on bull semen quality after cryopreservation. Span. J. Agric. Res..

[77] Ugur M.R., Saber Abdelrahman A., Evans H.C., Gilmore A.A., Hitit M., Arifiantini R.I., Purwantara B., Kaya A., Memili E. (2019). Advances in Cryopreservation of Bull Sperm. Front. Vet. Sci..

[78] Anton M., Martinet V., Dalgalarrondo M., Beaumal V., David-Briand E., Rabesona H. (2003). Chemical and structural characterisation of low-density lipoproteins purified from hen egg yolk. Food Chem..

[79] Holt W. (2000). Basic aspects of frozen storage of semen. Anim. Reprod. Sci..

[80] El-Sheshtawy R.I., Sisy G.A., El-Nattat W.S. (2015). Effects of different concentrations of sucrose or trehalose on the post-thawing quality of cattle Yak semen. Asian Pac. J. Reprod..

[81] Ijaz A., Hussain A., Aleem M., Yousaf M., Rehman H. (2009). Butylated hydroxytoluene inclusion in semen extender improves the post-thawed semen quality of Nili-Ravi buffalo (Bubalus bubalis). Theriogenology.

[82] Ansari M.S., Rakha B.A., Andrabi S.M., Ullah N., Iqbal R., Holt W.V., Akhter S. (2012). Glutathione-supplemented tris-citric acid extender improves the post-thaw quality and in vivo fertility of buffalo (Bubalus bubalis) bull spermatozoa. Reprod. Biol..

[83] Sarlós P., Molnár A., Kókai M., Al E. (2002). Comparative evaluation of the effect of antioxidants in the conservation of ram semen. Acta Veter- Hung..

[84] Li K.L., Lu J.H., Zhu X.S. (1986). Semen freezing and artificial insemination of semi-blood wild yak. J. China Yak.

[85] Campi S., Jorge A., Lombardo D., Blasi C., Gambarotta M. (2016). Yak (Bos Grunniens) sperm nuclei morphology, morphometry and DNA content. J. Dairy Vet. Anim. Res..

[86] Dvořáková K., Moore H.D.M., Šebková N., Paleček J. (2005). Cytoskeleton localization in the sperm head prior to fertilization. Reproduction.

[87] Peknicova J., Kubatova A., Sulimenko V., Draberova E., Viklicky V., Hozak P. (2001). Differential subcellular distribution of tubulin epitopes in Yak spermatozoa: Recognition of class III beta-tubulin epitope in sperm tail. Biol. Reprod..

[88] Romarowski A., Velasco Félix Á.G., Rodríguez P.T., Gervasi M.G., Xu X., Luque G., Contreras-Jiménez G., Sánchez-Cárdenas C., Ramirez-Gomez H.V., Krapf D. (2018). Super-resolution imaging of live sperm reveals dynamic changes of the actin cytoskeleton during acrosomal exocytosis. J. Cell Sci..

[89] Milardi D., Colussi C., Grande G., Vincenzoni F., Pierconti F., Mancini F., Baroni S., Castagnola M., Marana R., Pontecorvi A. (2018). Olfactory Receptors in Semen and in the Male Tract: From Proteome to Proteins. Front. Endocrinol..

[90] Kim S.W., Ki M.S., Kim C.-L., Hwang I.-S., Jeon I.S. (2017). A Simple Confocal Microscopy-based Method for Assessing Sperm Movement. Dev. Reprod..

[91] Moscatelli N., Spagnolo B., Pisanello M., Lemma E.D., De Vittorio M., Zara V., Pisanello F., Ferramosca A. (2017). Single-cell-based evaluation of sperm progressive motility via fluorescent assessment of mitochondria membrane potential. Sci. Rep..

[92] Zhao N., Chen S., Hong Y., Tang B.Z. (2015). A red emitting mitochondria-targeted AIE probe as an indicator for membrane potential and mouse sperm activity. Chem. Commun..

[93] Moretti E., Sutera G., Collodel G. (2016). The importance of transmission electron microscopy analysis of spermatozoa: Diagnostic applications and basic research. Syst. Biol. Reprod. Med..

[94] Coppola G., Di Caprio G., Wilding M., Ferraro P., Esposito G., Di Matteo L., Dale R., Dale B. (2013). Digital holographic microscopy for the evaluation of human sperm structure. Zygote.

[95] Di Caprio G., Gioffrè M.A., Saffioti N., Grilli S., Ferraro P., Puglisi R., Balduzzi D., Galli A., Coppola G. (2010). Quantitative Label-Free Animal Sperm Imaging by Means of Digital Holographic Microscopy. IEEE J. Sel. Top. Quantum Electron..

[96] Kemper B., Von Bally G. (2008). Digital holographic microscopy for live cell applications. Appl. Opt..

[97] Merola F., Miccio L., Memmolo P., Di Caprio G., Galli A., Puglisi R., Balduzzi D., Coppola G., Netti P., Ferraro P. (2013). Digital holography as a method for 3D imaging and estimating the biovolume of motile cells. Lab A Chip.

[98] Huser T., Orme C.A., Hollars C.W., Corzett M.H., Balhorn R. (2009). Raman spectroscopy of DNA packaging in individual human sperm cells distinguishes normal from abnormal cells. J. Biophotonics.

[99] Mallidis C., Wistuba J., Bleisteiner B., Damm O.S., Gross P., Wubbeling F., Fallnich C., Burger M., Schlatt S. (2011). In situ visualization of damaged DNA in human sperm by Raman microspectroscopy. Hum. Reprod..

[100] Edengeiser E., Meister K., Bründermann E., Büning S., Ebbinghaus S., Havenith M. (2015). Non-invasive chemical assessment of living human spermatozoa. RSC Adv..

[101] DE Angelis A., Managò S., Ferrara M.A., Napolitano M., Coppola G., De Luca A.C. (2017). Combined Raman Spectroscopy and Digital Holographic Microscopy for Sperm Cell Quality Analysis. J. Spectrosc..

[102] Ferrara M.A., De Angelis A., De Luca A.C., Coppola G., Dale B., Coppola G. (2015). Simultaneous Holographic Microscopy and Raman Spectroscopy Monitoring of Human Spermatozoa Photodegradation. IEEE J. Sel. Top. Quantum Electron..

[103] Holt W.V., O'Brien J., Abaigar T. (2007). Applications and interpretation of computer-assisted sperm analyses and sperm sorting methods in assisted breeding and comparative research. Reprod. Fertil. Dev..

[104] Alquézar-Baeta C., Gimeno-Martos S., Jiménez S.M., Santolaria P., Yániz J., Palacín I., Casao A., Cebrián-Pérez J., Muiño-Blanco T., Pérez-Pé R. (2019). OpenCASA: A new open-source and scalable tool for sperm quality analysis. PLOS Comput. Biol..

[105] Quintero-Moreno A., Miró J., Rigau A.T., Rodríguez-Gil J. (2003). Identification of sperm subpopulations with specific motility characteristics in stallion ejaculates. Theriogenology.

[106] Amann R.P., Waberski D. (2014). Computer-assisted sperm analysis (CASA): Capabilities and potential developments. Theriogenology.

[107] Givan A.L., Hanover N.H. (2011). Flow Cytometry: An Introduction.

[108] Cossarizza A., Chang H., Radbruch A., Akdis M., Andrä I., Annunziato F., Bacher P., Barnaba V., Battistini L., Bauer W.M. (2017). Guidelines for the use of flow cytometry and cell sorting in immunological studies. Eur. J. Immunol..

[109] Julius M.H., Masuda T., Herzenberg L.A. (1972). Demonstration That Antigen-Binding Cells Are Precursors of Antibody-Producing Cells After Purification with a Fluorescence-Activated Cell Sorter. Proc. Natl. Acad. Sci. USA.

[110] Althouse G., Hopkins S. (1995). Assessment of boar sperm viability using a combination of two fluorophores. Theriogenology.

[111] Matyus L., Szabo G., Resli I., Gaspar R., Damjanovich S. (1984). Flow cytometric analysis of viability of Yak sperm cells. Acta Biochim. Biophys. Acad. Sci. Hung..

[112] Kavak A., Johannisson A., Lundeheim N., Rodriguez-Martinez H., Aidnik M., Einarsson S. (2002). Evaluation of cryopreserved stallion semen from Tori and Estonian breeds using CASA and flow cytometry. Anim. Reprod. Sci..

[113] Wronski R., Golob N., Grygar E., Windisch M. (2002). Two-color, fluorescence-based microplate assay for apoptosis detection. BioTechniques.

[114] Peña F., Ball B.A., Squires E.L. (2017). A New Method for Evaluating Stallion Sperm Viability and Mitochondrial Membrane Potential in Fixed Semen Samples. Cytom. Part B Clin. Cytom..

[115] Kumaresan A., Johannisson A., Al-Essawe E.M., Morrell J.M. (2017). Sperm viability, reactive oxygen species, and DNA fragmentation index combined can discriminate between above- and below-average fertility bulls. J. Dairy Sci..

[116] Morrell J.M., Valeanu A.S., Lundeheim N., Johannisson A. (2018). Sperm quality in frozen beef and dairy bull semen. Acta Veter. Scand..

[117] Nagy S., Jansen J., Topper E.K., Gadella B.M. (2003). A Triple-Stain Flow Cytometric Method to Assess Plasma- and Acrosome-Membrane Integrity of Cryopreserved Bovine Sperm Immediately after Thawing in Presence of Egg-Yolk Particles1. Biol. Reprod..

[118] Alvarez M., Tamayo-Canul J., Anel E., Boixo J., Mata-Campuzano M., Martinez-Pastor F., Anel L., de Paz P. (2012). Sperm concentration at freezing affects post-thaw quality and fertility of ram semen. Theriogenology.

[119] Robles V., Martínez-Pastor F. (2012). Flow Cytometric Methods for Sperm Assessment. Spermatogenesis.

[120] Agarwal A., Virk G., Ong C., Du Plessis S.S. (2014). Effect of Oxidative Stress on Male Reproduction. World J. Men’s Health.

[121] Guthrie H.D., Welch G.R. (2006). Determination of intracellular reactive oxygen species and high mitochondrial membrane potential in Percoll-treated viable boar sperm using fluorescence-activated flow cytometry1. J. Anim. Sci..

[122] Wang H., Joseph J.A. (1999). Quantifying cellular oxidative stress by dichlorofluorescein assay using microplate reader. Free Radic. Biol. Med..

[123] Wardman P. (2007). Fluorescent and luminescent probes for measurement of oxidative and nitrosative species in cells and tissues: Progress, pitfalls, and prospects. Free. Radic. Biol. Med..

[124] Zielonka J., Vasquez-Vivar J., Kalyanaraman B. (2007). Detection of 2-hydroxyethidium in cellular systems: A unique marker product of superoxide and hydroethidine. Nat. Protoc..

[125] Evenson D., Jost L. (2000). Sperm chromatin structure assay is useful for fertility assessment. Methods Cell Sci..

[126] Benchaib M., Lornage J., Mazoyer C., Lejeune H., Salle B., Guerin J.F. (2007). Sperm deoxyribonucleic acid fragmentation as a prognostic indicator of assisted reproductive technology outcome. Fertil. Steril..

[127] Domínguez-Rebolledo Á.E., Fernández-Santos M., García-Álvarez O., Maroto-Morales A., Garde J., Martínez-Pastor F. (2009). Washing increases the susceptibility to exogenous oxidative stress in red deer spermatozoa. Theriogenology.

[128] Johnson L.A., Welch G.R., Rens W. (1999). The Beltsville sperm sexing technology: High-speed sperm sorting gives improved sperm output for in vitro fertilization and AI. J. Anim. Sci..

[129] Garner D.L. (2001). Sex-Sorting mammalian sperm: Concept to application in animals. J. Androl..

[130] Ge S., Garner D. (2002). Current status of sexing mammalian spermatozoa. Reproduction.

